# Effectiveness of an intervention to facilitate prompt referral to memory clinics in the United Kingdom: Cluster randomised controlled trial

**DOI:** 10.1371/journal.pmed.1002252

**Published:** 2017-03-14

**Authors:** Gill Livingston, Gianluca Baio, Andrew Sommerlad, Simon de Lusignan, Spyridon Poulimenos, Steve Morris, Greta Rait, Juanita Hoe

**Affiliations:** 1 Division of Psychiatry, University College London, London, United Kingdom; 2 Camden and Islington NHS Foundation Trust, London, United Kingdom; 3 Department of Statistical Science, University College London, London, United Kingdom; 4 Department of Clinical and Experimental Medicine, University of Surrey, Guildford, United Kingdom; 5 Department of Applied Health Research, University College London, London, United Kingdom; 6 Research Department of Primary Care and Population Sciences, University College London, London, United Kingdom; University of California San Francisco Memory and Aging Center, UNITED STATES

## Abstract

**Background:**

Most people with dementia do not receive timely diagnosis, preventing them from making informed plans about their future and accessing services. Many countries have a policy to increase timely diagnosis, but trials aimed at changing general practitioner (GP) practice have been unsuccessful. We aimed to assess whether a GP’s personal letter, with an evidence-based leaflet about overcoming barriers to accessing help for memory problems—aimed at empowering patients and families—increases timely dementia diagnosis and patient presentation to general practice.

**Methods and finding:**

Multicentre, cluster-randomised controlled trial with raters masked to an online computer-generated randomisation system assessing 1 y outcome. We recruited 22 general practices (August 2013–September 2014) and 13 corresponding secondary care memory services in London, Hertfordshire, and Essex, United Kingdom. Eligible patients were aged ≥70 y, without a known diagnosis of dementia, living in their own homes. There were 6,387 such patients in 11 intervention practices and 8,171 in the control practices. The primary outcome was cognitive severity on Mini Mental State Examination (MMSE). Main secondary outcomes were proportion of patients consulting their GP with suspected memory disorders and proportion of those referred to memory clinics. There was no between-group difference in cognitive severity at diagnosis (99 intervention, mean MMSE = 22.04, 95% confidence intervals (CIs) = 20.95 to 23.13; 124 control, mean MMSE = 22.59, 95% CI = 21.58 to 23.6; *p* = 0.48). GP consultations with patients with suspected memory disorders increased in intervention versus control group (odds ratio = 1.41; 95% CI = 1.28, 1.54). There was no between-group difference in the proportions of patients referred to memory clinics (166, 2.5%; 220, 2.7%; *p* = .077 respectively). The study was limited as we do not know whether the additional patients presenting to GPs had objective as well as subjective memory problems and therefore should have been referred. In addition, we aimed to empower patients but did not do anything to change GP practice.

**Conclusions:**

Our intervention to access timely dementia diagnosis resulted in more patients presenting to GPs with memory problems, but no diagnoses increase. We are uncertain as to the reason for this and do not know whether empowering the public and targeting GPs would have resulted in a successful intervention. Future interventions should be targeted at both patients and GPs.

**Trial registration:**

Current Controlled Trials ISRCTN19216873

## Abstract 2

Gill Livingston and colleagues present results of a cluster randomized controlled trial of an intervention aimed to facilitate prompt referral to memory clinics and increase early diagnosis of dementia in the United Kingdom.

## Author summary

**Why was this study done?:**

- Early (timely) diagnosis of dementia allows people to plan for the future, receive symptomatic treatment, access social and voluntary care, helps family carers, reduces crises, and delays care home entry for people with dementia with little negative effects.
- Many countries have a policy to increase timely diagnosis, but trials aimed at changing general practitioner (GP) behaviour have been unsuccessful.
- We aimed to assess whether empowering patients and their families by sending patients aged 70 or over without a known diagnosis of dementia the intervention (a GP’s personal letter, with an evidence-based leaflet about overcoming barriers to accessing help for memory problems) increases earlier dementia diagnosis through GPs referral to memory clinics compared to continuing usual practice alone (control).

**What did the researchers do and find?:**

- GP practices were randomly allocated to send patients a personally signed letter and information leaflets (11 practices, 6,387 patients) or usual care (11 practices, 8,171 patients).
- Our intervention had no effect at increasing timely or overall diagnosis as the memory scores (measured by Mini Mental State Examination) of people in the intervention and the control group (22.04 versus 22.59) at diagnosis were not statistically, nor substantially, different.
- There was a significant increase in the proportion of patients in the intervention practices compared to control practice consulting their GPs with suspected memory difficulties (odds ratio = 1.4; 95% CI = 1.28, 1.54) but no difference in the proportion of all practice patients referred to memory services (intervention group = 2.5% versus control group = 2.7%).

**What do these findings mean?:**

- Empowering patients using a GP’s personal letter plus evidence-based leaflet is not by itself enough to improve timely access to dementia services.
- Our study was limited, as we do not know whether these extra patients were more concerned, but without memory problems “worried well,” or whether they had memory problems which were not recognised by their GP.
- Future interventions could target both public and practitioners, concentrating on earlier diagnosis benefits.

## Introduction

The number of people with dementia is increasing worldwide as the population ages [1]. Across developed countries, many people with dementia never receive a diagnosis, while others receive one late in the illness [2]. Our systematic review of interventions to increase the rates of dementia diagnosis reports no clearly successful intervention; although educating general practitioners (GPs) increased their ability to diagnose dementia, this approach did not result in increased diagnostic rates [3]. Currently, the dementias are incurable, but early diagnosis allows people to plan for the future [4], receive treatment to reduce cognitive and neuropsychiatric symptoms [5,6], and access social and voluntary care. Early diagnosis also helps family carers [7], reduces crises, and delays care home entry [8] for people with dementia with little negative effects [9].

Family carers report difficulty in obtaining a diagnosis of dementia for their relative, which can take several years, with the delay causing increased anxiety and carer burden [10–12]. Families find that some relatives with memory problems are reluctant to consult their GP about it and deny problems when seen [11,13]. Barriers to seeking help or diagnosis include fear of the diagnosis, concerns about stigma, GP disinclination to make this diagnosis, negative responses from other family members, normalisation of symptoms, and a lack of awareness about the signs of dementia [13–16].

Our systematic review of interventions to increase the rates of dementia diagnosis reports no clearly successful intervention; although educating GPs increased their ability to diagnose dementia, this did not result in increased diagnostic rates [3]. Previous interventions have neither specifically targeted and tried to empower older people nor used the relationship with GPs to address barriers to diagnosis other than symptom recognition. This has helped in other fields; for example, GP personal letters with information leaflets changed patient’s behaviour regarding diagnosis of bowel cancer, leading to increase in uptake of diagnostic procedures [17].

We therefore aimed to facilitate patients and families to present to their GP and gain a timely diagnosis of dementia (as evaluated through cognitive severity at presentation), through a personalised letter and evidence-based leaflet [11] sent directly to patients registered with general practices and to evaluate this in a randomised controlled trial (RCT).

## Methods

### Study design

Multicentre, parallel group, cluster RCT. We recruited general practices in southeast England (north and east London, Hertfordshire, and Essex). The practices were diverse in socioeconomic status and ethnic composition and ranged from inner-city to rural locations. The corresponding memory services were in Camden and Islington NHS Foundation Trust, North East London NHS Foundation Trust, East London NHS Foundation Trust, Barnet, Enfield and Haringey Mental Health Trust, and Hertfordshire NHS Partnership Foundation Trust. We obtained written ethics approval for the study from National Research Ethics Service (NRES) Committee London, Queen Square for the trial (ID: 13\LO\0996). Research and development permission was obtained from local trusts and clinical commissioning group areas in which the GP practices were located. The protocol is available (S1 Text) at http://www.isrctn.com/ISRCTN19216873 and the CONSORT statement in S2 Text.

### Patients

We included registered patients 70 y or older within general practices. We excluded people known to have dementia or who lived in care homes. All participating GP practices and memory clinics gave written informed consent.

### Randomisation and masking

The trial statistician (GB) set up an online computer-generated randomisation system, allocating participants to intervention or usual care in ratio 1:1 stratified by geographical location (London, Essex, Hertfordshire) using random permuted blocks. He was not involved in the remainder of the trial until analysis. Independent raters, masked to randomisation status, collected data about patients referred from participating GP practices to memory services, both for the year before and the year after the study. We masked memory services but could not mask GP practices or their patients who received a leaflet. Masked raters collected information from Morbidity Information Query and Export Syntax (MIQUEST), a Department of Health interface for anonymised data extraction from GP practices [18].

### Procedures

#### Recruitment and follow-up

We recruited GP practices and the corresponding memory services to which GP practices referred and collected anonymised baseline and follow-up data. Cluster Caldicott guardians gave consent to collect anonymised data and for the RCT [19] as gatekeepers of patient groups when consent must be given for a whole cluster.

#### Intervention

The original leaflet was developed from a study with family carers about barriers and facilitators to seeking help for memory problems [11]. We used the UK Medical Research Council (MRC) complex intervention development recommendations [20] to improve the intervention through consultation with the Alzheimer’s Society GP reference group, family carers, people with mild cognitive impairment (MCI), and dementia experts. We refined the text content, layout, colour, illustrations, usability, and acceptability. We then used a professional designer. The leaflet contains information about overcoming common barriers to accessing dementia diagnosis and care if people are concerned about themselves or a relative. It covers how to persuade someone to go to the doctor to seek help if there are memory worries, what information to give to the GP, how to be referred to specialist services, confidentiality, overcoming refusal of help, information available for patients and families about dementia, and what to do if things are not working.

We developed a personal letter signed by their GP to the “at risk” population (people ≥70 y living at home without a known diagnosis of dementia) to accompany the leaflet, following similar consultation. The letter was addressed to the individual patient and explained the leaflet was about how to get help for themselves or someone else they knew with memory problems. It outlined potential symptoms, stressed these should have persisted for over 3 mo, that help is available, and they should contact their GP if worried about themselves or a relative. The GPs did not change the main text of the letter, but they provided personalised contact details. Leaflet and letter are in S3 and S4 Text and the leaflet can be found at http://www.ucl.ac.uk/psychiatry/research/olderpeople.

#### Treatment as usual

The control group received usual care in line with standard current practice. Patients presented to the GP as they wished and GPs assessed and considered a memory service referral for diagnostic assessment. There are current clinical guidelines for dementia care [12].

#### Assessments

We collected the following information from memory services for the year before and after baseline:

1 Sociodemographic data: sex, age, ethnic status according to census, marital status, education.
2 Cognitive score at diagnosis; measured by the Mini Mental State Examination [21] (MMSE) or the Addenbrooke’s Cognitive Examination (ACE-R or ACE-III) [22,23] of people who received a diagnosis of dementia or MCI.
3 The number of patients referred to memory services from intervention and control practices.
4 The number of memory services appointments offered and people attending for diagnostic assessment.

Possible adverse events:

5 The number of people referred who were assessed as not having dementia or MCI.
6 We asked at the commencement of the study for every GP practice to inform us, and then checked after, about inappropriate presentations, patient distress, and whether the intervention led to unacceptable numbers of presentations.

We collected the following information from GPs for the year before and after baseline:

1. We recorded the number of eligible patients in each practice.
2. Memory, cognitive, or dementia-related examinations. We used MIQUEST (a data extraction tool, which allows the anonymous extraction of patient information about the content of every clinical consultation from the different brands of GP computerised medical record systems) to extract data from GP notes. We prespecified codes encompassing cognitive examination or a comment about memory, cognitive, or dementia screening, assessment, testing, observation, level or reviewing. We collected whether each patient aged over 50-y-old had one or more consultation about their memory over the year after randomisation. As the data were anonymised, we could not link it to patients referred to memory clinics.

### Outcomes

Our primary outcome was to determine if our intervention led to people with dementia or MCI presenting earlier to specialist dementia services, i.e., with higher cognitive scores at diagnosis compared with usual care over 12 mo.

The secondary outcomes compared the effect of the intervention versus treatment as usual (TAU) on:

1 Number of eligible patients presenting with cognitive problems to their GP.
2 Rate of GP referral of patients with a suspected diagnosis of dementia to memory services.
3 The proportion of eligible patients who were referred to a memory clinic.
4 The number of patients subsequently diagnosed with a cognitive disorder (dementia or MCI).
5 The number of memory service appointments offered to those referred.
6 The costs of implementing the intervention.

Possible harm:

7 GP referral of those who do not have dementia or MCI (as the intervention may have caused worry in those who are well).
8 GP practice report of negative comments from patients or relatives sent the interventions.

### Patient involvement

Family carers were interviewed for the original content of the leaflet [11] and then family carers, people with cognitive disorder, and GPs from the Alzheimer’s Society were asked about leaflet redesign. Members of the Alzheimer’s Society Research Network contributed to the design of the study, monitored the conduct of the study, and were on the steering group.

### Statistical analysis

#### Power calculation

This sample size was based on the average GP-registered patient population of 2,000, of whom 260 (13%) were aged 70 y or older [24]. An audit of the referral rates and MMSE scores of people with dementia presenting to one memory service found the mean number of referrals was 11 patients/ practice/y, with a mean MMSE = 19.5 (standard deviation, SD = 6.1). A difference of three points in MMSE has been reported as clinically significant [25]. We required 71 undiagnosed people with dementia to present to memory clinics in each group in order to detect a difference of three points, with 90% power at a 5% significance level. To account for clustering within each GP practice, we inflated the sample based on a projected intracluster correlation (ICC) of 0.03 [26]. Based on these calculations, we required a sample size of 93 patients referred in each group. We allowed for 18% attrition of referred patients who did not have the full memory clinic assessment, thus inflating the sample size to 114/group. We planned to recruit 11 GP practices to each arm of the study using the estimate of 11 patients referred per practice per year.

#### Changes to trial outcome after the trial commenced

The MMSE was the measure of cognition we had envisaged being available for most patients. However, possibly because of a change in charging for the use of the MMSE [27], many memory services were using other cognitive measures, most commonly the 100 point ACE-R and ACE-III. We decided before we began the analysis to include patients with MMSE scores and to make a complete dataset of MMSE for those without MMSE scores but with ACE scores by imputing the missing data. We employed multiple imputation after using data on the latter to estimate the missing MMSE scores. We ran a regression model on the 221 patients across both study arms who had both MMSE and ACE. This included some people who were excluded from the analysis of outcomes, as they were already known to have dementia.

The MMSE score was the response variable and the ACE score the predictor. The coefficients of the intercept and the ACE score were statistically significant and consistent with a recent study of established dementia [28]. This latter model assumes absence of the MMSE is explained entirely by the ACE value, which may be too simplistic. We therefore used multiple imputation by chained equations (MICE) [29] and controlled for other potentially relevant factors: sex, age, marital status, living condition, education, area-level index of multiple deprivation, and a random effect by practice. Following standard practice, we imputed five complete datasets and analysed them separately, pooling the results using Rubin’s rule [30] and using these estimates for the remaining analysis. We then converted the ACE scores using the ACE and MMSE as a single outcome score (called “combined MMSE” in this paper).

#### Analysis

There was no study data monitoring committee. We reported new referrals data of those with cognitive disorders by randomisation group. We compared the mean combined MMSE from each group, using *t* tests to assess statistical differences controlling for age, education, and sex.

We used hierarchical multivariate regression analysis to account for patient clustering and to adjust for baseline differences in outcomes. We used multiple linear regression to identify demographic predictors of combined MMSE at presentation: sex, age, education, and ethnicity. Random effects were included to account for the clustering effect of GP practices. We adjusted for potential missing data using multiple imputations. We also repeated this in a sensitivity analysis of only those patients who had MMSE scores.

We performed chi-square tests comparing the proportion of referrals to memory services between trial arms and between the year before and the year after the intervention.

We compared GP contacts from MIQUEST [18] practice records using a regression model and accounting for recording of memory assessments the year before baseline.

We calculated the costs of the intervention based on the costs of leaflet printing, of identifying patients to send it to, and of postage and packing.

#### Post hoc analysis

As the study covered years in which increasing the timely diagnosis of dementia had become a national priority, we performed a post hoc analysis of change in numbers of those referred, diagnosis of cognitive disorder, and stage of referral (cognitive score) in both groups over the year before and after the intervention.

All statistical analyses followed a predefined analysis plan and were carried out using the freely available statistical software R, version 3.2.0.

## Results

**Fig 1:**
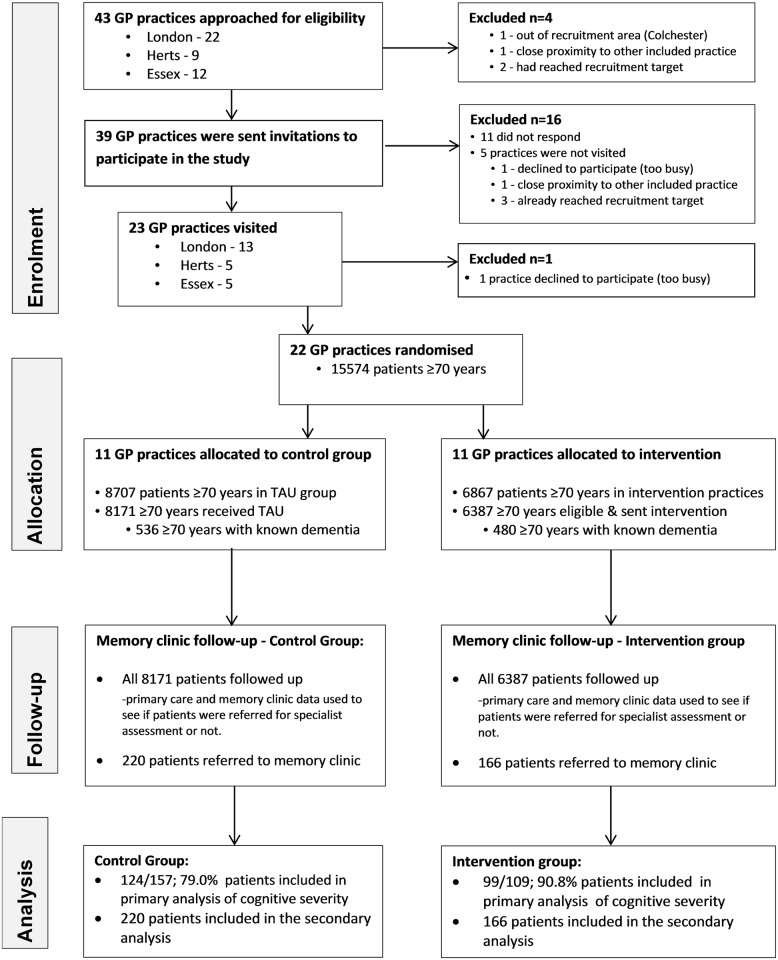
CONSORT flow diagram.

We approached 43 practices, and 22 were randomised. The consolidated reporting of trials (CONSORT: Fig 1) diagram shows practices’ and patients’ progress through the trial. We recruited GP practices between 16th August 2013 and 14th December 2013 and the corresponding memory services between 11th December 2013 and 23rd September 2014. All memory services linked to the practices included in the study were recruited. We collected follow-up data from participating memory services between 16th April 2014 and 2nd October 2015. We stopped the trial once we had recruited the practices and follow-up was completed. Table 1 shows the baseline characteristics of those presenting to memory clinics with a suspected diagnosis of dementia. As expected, the majority of patients were female and the mean age was around 80-y-old.

**Table 1:** Demographic characteristics of those presenting to memory clinics with suspected dementia.

Demographic		Intervention	Control	All
**Gender (%)**	Male	76 (45.8) *n* = 166	84 (38.2) *n* = 220	160 (41.5) *n* = 386
**Age** *n* = 386	Mean; SD (range)	80.52; 8.97 (47–97) *n* = 166	79.78; 8.60 (51–97) *n* = 220	80.10; 8.76 (47–97)
**Ethnicity (%)** *n* = 302	White	85 (76.6)	173 (90.6)	258 (85.4)
	British other	26 (23.4)	18 (9.4)	44 (14.6)
**Marital status (%)** *n* = 350	Currently not living with partner/married	77 (52.7)	98(48.3)	175 (50)
	Currently married/living with partner	70 (47.6)	105 (51.7)	175 (50)
**Living situation (%)** *n* = 353	Living alone	76 (49.0)	84 (42.4)	160 (45.3)
	Living with other	79 (50.1)	114 (57.6)	193 (54.7)
**Level of education (%)** *n* = 262	No education	2 (1.9)	2 (1.3)	4 (1.5
	Primary	2 (1.9)	2 (1.3)	4 (1.5)
	Secondary	82 (76.6)	107 (69.0)	189 (72.1)
	Post–secondary education	21 (19.6)	44 (28.4)	65 (24.8)

SD, standard deviation

There were 223 people with scores from the combined Mini Mental State Examination (MMSE) at follow-up (99/109; 90.8% in the intervention and 124/157; 79.0% control), of whom 173 had had an MMSE; 83 intervention and 90 control. The intracluster correlation (ICC) for the GP practices for the primary outcome was 0.0024. Three hundred and eighty-six patients were referred but because of death, refusal, and moving, 62 were never fully assessed. Two hundred and sixty-six out of three hundred and twenty-four people (82.15%) who presented to memory clinics from the practices and were fully assessed received a diagnosis of cognitive disorder.

Overall, we analysed 223/266 (83.8%) diagnosed patients. The raw scores on MMSE and Addenbrooke’s Cognitive Examination (ACE) in each group are in Table 2. There was no effect of the intervention on cognitive severity at diagnosis (intervention group mean combined MMSE score = 22.04, 95% CI = 20.95 to 23.13, standard deviation (SD) = 5.54; control group mean combined MMSE = 22.59, 95% CI = 21.58 to 23.6, SD = 5.73; *p* = 0.48).

**Table 2:** MMSE and ACE scores.

	Control				Intervention			
	N	Mean	SD	range	N	Mean	SD	range
MMSE scores	90	22.3	6.3	0–30	83	22.4	5.5	0–29
ACE	83	69.2	16.2	30–96	67	65.5	16.5	22–92

Similarly, in a sensitivity analysis, we found no evidence of an effect of the intervention on the mean MMSE at presentation between the 83 patients in the intervention (mean MMSE score: 22.4, SD: 5.5) and 90 in the control group (mean MMSE score: 22.3, SD: 6.3) with a valid MMSE (*p* = 0.91).

Both intervention and control groups had more presentations to the GP for cognitive problems in the year after than the year before baseline (see Table 3 for secondary outcomes. There were more consultations with people with a suspected memory disorder in the GP practices in the intervention compared to the control adjusted for the initial rate of GP recording. GPs who had recorded seeing more patients with memory problems in the year before baseline also recorded seeing more in the year after. Both intervention and control groups present more to their GP with suspected memory problems although this increase is greater in those in the intervention than the control group (odds ratio [OR] = 1.41, CI: 1.28–1.54). GPs reacted to the increased numbers presenting to them by referring a lesser proportion than in the year before the intervention, and the decrease is more marked in the intervention group (chance of being referred in intervention group versus control OR = 0.70, CI: 0.57–0.85). However, as more patients presented to their GP, the proportion of eligible patients (those aged over 70) referred to a memory clinic increased in both groups and there was no significant difference between the two groups (OR = 1.08; CI: 0.91–1.27).

**Table 3:** Secondary outcomes for intervention groups versus control in the year after intervention.

	Intervention	Control	Odds ratio
	Number/eligible population, %	Number/eligible population, %	*Intervention versus control
Percentage of eligible patients presenting with cognitive problems to their GP (patients aged ≥70-y-old)	Preintervention 293/6,387 (4.6%)	Preintervention 223/8,171 (2.8%)	OR = 1.41, CI: 1.28–1.54
	Postintervention 699/6,387 (10.9%);	Postintervention 699/8,171 (8.55%)	
Patients referred to memory clinics/number of patients presenting to their GPs with memory problems (controlling for baseline patients aged ≥70 y	Preintervention 106/293 (36.18%);	Preintervention 104/223 (46.64%)	OR = 0.70, CI: 0.57–0.85
	Postintervention 166/699 (23.75%);	Postintervention 220/699 (31.47%)	
Patients referred to memory clinics/number eligible patients in the group (controlling for baseline)	Preintervention 106/6,387 (1.66%);	Preintervention 104/8,171 (1.27%)	OR = 1.08, CI: 0.91–1.27
	Postintervention 166/6,387 (2.6%);	Postintervention 220/8171 (2.69%)	
The number of patients subsequently diagnosed with a cognitive disorder of those assessed (dementia or MCI).	109/132 (82.6%) 90 dementias 19 MCI	157/192(81.8%) 108 dementia 49 MCI	
Number of memory service appointments offered to those referred	160/166 (96.4%)	206/220 (93.6%)	
GP referral of those who do not have dementia or MCI	23 (34 more not completely assessed)	35 (28 more not completely assessed)	
The costs of implementing the intervention.	£2.16	£0.00	
GP practice report of negative comments from patients or relatives sent the interventions	0	0	

*Controlled for baseline

MCI, mild cognitive impairment

There was no between-group difference in the rate of referral in the year after the intervention of eligible patients in the practices (2.5% and 2.7% intervention and control group, respectively). Nor was there a between-group difference in referral rate of those subsequently diagnosed with either dementia or mild cognitive impairment (MCI; 113: 85.6% and 161: 83.9%; *p* = 0.79 for intervention and control group, respectively). Both groups had significant increase in diagnosis rates of cognitive disorder in memory clinics between the year before baseline and follow-up: the intervention arm increased from 16.2% to 22.6%; and for control from 12.0% to 23.0%, overall *p* < 0.0001.

There was no difference in the rate of memory services appointments of eligible patients offered in the year after the intervention between the intervention and the control group (160/6,387 [2.5%] and 206/8,171 [2.5%], respectively [*p* = 0.99]).

Both intervention and control groups had a significant increase in referrals to memory services between the year before baseline and the year after. The intervention arm increased from 106 (1.65%) to 166 (2.6%), *p* < 0.0003; and for control from 104 (1.3%) to 220 (2.7%), *p* < 0.0001. For both arms combined, the preintervention and postintervention rates were 210 (1.4%) and 386 (2.6%; *p* < 0.0001), respectively. The mean combined MMSE score for the whole population in the year before the intervention was 21.5, and the year after was 22.1 (*p* = 0.38).

There was no difference between the intervention and the control group in the rate of people referred without a cognitive disorder in the year after the intervention; 23 (0.3%) versus 35 (0.4%; *p* = 0.61), although this increased in both groups over time; intervention group (0.2% to 0.3%; *p* = 0.19); control group (0.1% to 0.4%; *p* < 0.0004).

All GPs were contacted, and no adverse events from the intervention were reported in relation to patient distress or difficulty with increased volume of work.

The costs of the leaflet were £2.16 per recipient, comprising £0.66 for producing the leaflet (£198 per 300 leaflets); £1 per patient to identify patients on practice lists to send the leaflet to, including identifying their postal address; and £0.50 for postage and packing costs.

## Discussion

There was no between-group difference in our primary outcome, cognitive severity at diagnosis. Our intervention was designed to empower patients and increase early diagnosis of dementia through increasing presentation to GPs with memory symptoms. The letters resulted in more people presenting to their GPs from the intervention practices with suspected memory problems. Although there was also a secular increase in GP referrals in both groups, GPs in the intervention group referred less of the people who presented to them with memory problems than in the nonintervention group. We do not know whether the extra patients presenting but not referred were worried well or had cognitive deficits, but overall in both groups there was no increase in the MMSE at diagnosis over time, so GPs are not referring earlier.

While very low cost, this evidence-based complex intervention planned in accordance with the Medical Research Council (MRC) complex intervention development recommendations [20], and incorporating the identified elements of behaviour change of capability; opportunity; and motivation for the patients [31], joins the list of unsuccessful interventions to change GP behaviour and increase timely diagnosis. Previous interventions had not succeeded by trying to work through GPs, we therefore adopted a new approach.

We are unable to be definitive as to why our approach was unsuccessful, but there are several possible reasons. Our intervention helped empower patients with memory problems to present to GPs. There was nothing specifically targeted at GPs to change their referral behaviour. The intervention may have encouraged some people who do not have dementia to attend their GP and then be reassured their memory problem was not due to dementia, or receive treatment for another problem. Neither GPs nor their patients reported the intervention was upsetting or worrying or led to too much GP work. GPs may usually refer people at crisis points or with more severe dementia, and both GPs and some memory clinics, despite national policy, may not think referral is indicated in mild dementia. This seems to have been a pattern in the past with high mortality in the year after diagnosis [32].

While the national strategy to increase dementia diagnosis has changed the behaviour of GPs, no intervention trial has done so [3]. To our knowledge, this is the first analysis of whether the change in policy also leads to an earlier diagnosis in terms of cognitive severity. We find that it does not. Thus, GPs are referring more frequently but not earlier in the illness. It is possible that patients in the intervention group, who presented to GPs and were not referred, had a milder dementia or MCI.

Nonetheless, in the UK, an increase in diagnostic rates has followed the National Dementia Strategy and the subsequent increase in memory clinics [33]. The Dementia Identification Scheme, offering financial incentives to GPs for new diagnoses of dementia, started in October 2014 (https://www.england.nhs.uk/wp-content/uploads/2014/10/dementia-ident-schm-fin.pdf) and coincided with the study period. National statistics about the people on dementia registers and our finding of highly significant increases in referral rates since the year before baseline in both groups suggest the successful drivers of behaviour change were multifactorial and likely to include national strategies (UK National Dementia Strategy and the Prime Minister’s challenge on dementia) and societal change with an increase in publicity about dementia in the national media, which can be considered as large and continuing interventions.

GPs do not refer all patients with suspected dementia to memory clinics, but our data accounted for individual GPs’ previous referral practice. We did not expect the reduction in using the MMSE as an initial measure of cognition and had to modify our primary outcome measure. However, we successfully recruited to this study and there were enough people with data on the combined MMSE, with more than twice as many new referrals in each group than our initial power calculation required. We estimated missing MMSE scores by using the ACE score and other covariates. We assumed that MMSE score can be calculated by the observed covariates through our model and accounting for uncertainty in the estimation procedure using multiple imputation. While this is more robust than relating the MMSE and ACE scores using simple regression models to calculate point estimates, there may be unobserved factors, potentially explaining absence in the main outcome variable. We assumed the ACE-R and ACE111 were equivalent, as most of the questions are the same and they correlate significantly (r = 0.99, *p* < 0.01). The ACE-III also continues to show high sensitivity and specificity at cutoffs of the ACE-R [23]. We were, however, reassured that our other analyses produced similar results to our primary analysis. We equated timely referral with earlier referral, but this may not be so. The increase in diagnoses over time may mean more people were helped by an early referral. We did not undertake a full economic evaluation of the intervention.

There was considerable missing data regarding ethnicity from the practices in Hertfordshire, which are areas of around 85% white UK ethnicity for the whole population (http://www.theguardian.com/news/datablog/2011/may/18/ethnic-population-england-wales#data, accessed 23.6.16). The people in older age groups are probably even more predominantly of white UK ethnicity. Only two people whose ethnicity was reported from these areas were specified as being of nonwhite UK origin. Thus, 68/84 (81.1%) of the people whom we did not have ethnicity data came from these Hertfordshire areas. Our experience is that when an area’s population is overwhelmingly white UK, practitioners do not judge it necessary to specify those data in the notes. As other demographic data were detailed, this seems a likely explanation. If we assumed that those whose data were missing from any practice were white UK ethnicity, then 140 (84.3%) people in the intervention and 160 (92.3%) in the control were white UK, although this would probably be a slight overestimate.

While those who are forgetful are less likely to remember to respond to a letter, we were looking for people with early illness, who do not have an all-pervasive memory problem. In addition, the letter discussed whether they or another relative had a memory problem and could have been picked up by partners or other relatives they were living with. More than half the people referred lived with someone else.

We categorised the GP datasets through the coded data extracted using MIQUEST, but different GPs may record such data inconsistently, if at all. We were able to consider recording the year before the intervention and adjust for it. We therefore think the between-group differences are true differences.

### Clinical implications and conclusions

An evidence-based complex intervention to empower patients with memory symptoms or their families and increase early diagnosis of dementia through facilitating presentation to GPs was unsuccessful. It increased those with suspected memory problems presenting to their GPs but not referrals from there to memory services. We think this may be because GPs are concerned about the availability of services, both in terms of waiting lists for diagnostic services and of very limited postdiagnostic services.

We recruited from diverse areas and had excellent follow-up rates, so our findings of lack of effectiveness are likely to be generalisable that GPs are not referring patients earlier in the illness. Interventions likely to be successful in decreasing cognitive severity at diagnosis will need to target both the public and practitioners and particularly concentrate on the benefits of earlier diagnosis.

## Supporting information

S1 Text Trial protocol. (DOC)

S2 Text CONSORT statement. (DOC)

S3 Text GP intervention leaflet. (PDF)

S4 Text GP intervention letter. (PDF)

## Abbreviations

ACE: Addenbrooke’s Cognitive Examination
GP: general practitioner
ICC: intracluster correlation
MCI: mild cognitive impairment
MMSE: Mini Mental State Examination
MRC: Medical Research Council
OR: odds ratio
RCT: randomised controlled trial
SD: standard deviation
