# Supplementary material for: Effectiveness of an intervention to facilitate prompt referral to memory clinics in the United Kingdom: Cluster randomised controlled trial
Source: PLoS Med. 2017 Mar 14;14(3):e1002252. doi: 10.1371/journal.pmed.1002252 (PMC5349651; doi:10.1371/journal.pmed.1002252)
Supplement: S1 Text — (DOC) [file pmed.1002252.s001.doc]

### Scientific proposal: Facilitating Early Diagnosis of Dementia (FED-D)

**Sponsor:** UCL

**Chief Investigator:** Prof Gill Livingston

**Protocol version:** FED_D protocol_v2_220513.doc Date: 22/05/13

**1. Aims of the project**

The primary aim of this study is to increase timely diagnosis of dementia through an evidence-based intervention promoting memory service access. We will test in a randomised controlled trial if this intervention increases the number of people with undiagnosed dementia presenting with memory related problems to their GP; the number of patients GPs refer appropriately to memory services; and if this referral is at an earlier stage. We are currently collaborating with the Alzheimer’s Society and their reference groups (including the GP reference group) to redesign the evidence-based CHOICE leaflet^1^ to ensure usability, acceptability and relevance, and to develop a personal letter from GPs to the “at risk” population to accompany the leaflet.

Research questions

Does sending a leaflet^1^ on how to overcome barriers to accessing help for dementia to people aged over 70 registered in a general practice, with an accompanying personal letter from the GP, lead to people with dementia presenting earlier to specialist dementia services compared with usual care over 12 months, and if so is it at low cost? We will test the hypotheses that over 12 months people receiving the intervention will present earlier (mean of 3 points higher on MMSE score)^2^ than those not receiving it and that the costs of the intervention are small.

Primary objective

To enable people with dementia to receive an earlier diagnosis

Secondary objectives

1. To increase the number of people with dementia receiving diagnoses
2. To increase the numbers of people with memory problems presenting to GPs.
3. To calculate the cost-effectiveness of the intervention considering the cost of implementing the CHOICE intervention and the numbers of appropriate and inappropriate referrals to memory services.

**2. Work which has led up to the project**

Why early diagnosis of dementia needs to be increased

The number of people aged >65 will increase by 15%, and those >85 years by 27%, over the next decade^3^. Thus the 820,000 people currently living with dementia in the UK^4^ is rising, as dementia prevalence doubles with every five-year increase in age-band^5,6^. The UK Dementia Strategy^7^ promotes timely diagnosis (diagnosis made earlier in the disease trajectory) of dementia.

Family carers often experience difficulty in obtaining a diagnosis of dementia for their relative, which can take several years, and the delay results in increased anxiety and carer burden^7,8,9^. Families report that relatives with memory problems are reluctant to consult their GP about their memory and deny problems when seen^1^. Other barriers to seeking help include fear of the diagnosis, concerns about stigma, GP reluctance to make this diagnosis, and negative responses from other family members^1,7,10,11^.

People with dementia and carers usually feel relieved by diagnostic certainty and can access treatment with potentially better results, start to plan and make choices, and play a more active role in their current and future health care^1,11,12^. Early diagnosis and intervention in dementia facilitates access to specialist services, support and treatment, and is cost-effective^9,10,13^, reducing crises and delaying care-home admission^9,13^. Memory services have positive effects on the quality of life of people with dementia and treat neuropsychiatric symptoms which are the most common predictors of institutionalisation^13,14,15^. Care-home placement costs the UK £7 billion/year, with two-thirds paid for by tax and one-third in private funding^6,10,13^. The Croydon Memory Service model estimates that a 10% reduction in care home admissions over 10 years, through improving access to memory services for early dementia diagnosis and intervention, could result in savings of £120 million in health and social care expenditure and £125 million in private expenditure per year, with gains of 6,250 QALYS^13^.

The All-Party Parliamentary Group on Dementia (APPG)^16^ found that despite the emphasis in UK national policy’s on early and increased diagnosis in dementia, there has been little progress over the last few years^17^, comparing poorly with other European countries^10,18^. Conversely, huge geographical variation exists within the UK for diagnostic rates in dementia, suggesting it is possible to increase diagnosis^19^.

Previous work to increase early diagnosis of dementia

We are building on earlier work which improved GP knowledge about dementia and specialist services^7,8,9,10^. GPs know about dementia, but may lack experience and confidence in diagnosing it and informing patients^8,9,10,20^. Despite attempts, including education and financial incentives, to improve diagnosis of dementia in UK primary care, detection remains low even after presentation^9,10,21^, partly as early diagnosis can be difficult^23,24^. Community based campaigns about seeking help for memory problems are insufficient by themselves to ensure adequate early diagnosis of dementia^25^. The Alzheimer’s Society previously funded a community based leaflet campaign to increase awareness and presentation of people with dementia to GPs, which was unsuccessful in increasing diagnosis. Previous interventions have neither specifically targeted older people nor engaged the GP, nor used the relationship, nor addressed barriers to diagnosis other than symptom recognition.

The current intervention

Our intervention follows the Medical Research Council guidelines for development and evaluation of complex interventions^26,27^. We are building on peer reviewed, published qualitative work which identified the patient and healthcare barriers to early presentation with dementia and developed an intervention to engage patients and carers to improve early presentation^1^. Our work translates research findings into practice and tests the implementation of an existing evidence-based intervention. The intervention is currently being further developed by us in collaboration with the Alzheimer’s Society GP reference group and staff including dementia advisors; people with dementia patients and family carers and will be ready for testing if this grant is funded. These leaflets remain available to all^28^, but our intervention will target people to whom they are relevant and ensure they receive them. It aims to help both the person with memory problems and their families to overcome common barriers to seeing their GP after identifying symptoms of dementia. In addition, it delineates strategies to encourage GPs to refer the person with memory problems on for specialist assessment, where appropriate, in response to a relative’s or patient’s concerns about a patient’s memory loss.

GP personal letters with information leaflets changed patient’s behaviour in the field of cancer. In bowel cancer, such letters led to a massive increase in uptake of a difficult screen, involving a self administered enema at home, followed by endoscopy at clinic^29^. This study will test these methods, which have been successfully implemented in cancer research to raise awareness and increase presentation within the general population. Cancer and dementia are both terminal diseases in which early detection and diagnosis allow access to treatment and services. In cancer, early detection and treatment can be curative, or increase lifespan. While currently dementia is incurable, early diagnosis means the ability to plan ahead^11,12^, to receive treatment to reduce the cognitive and neuropsychiatric symptoms associated with dementia, improve quality of life^7^ and delay institutionalisation^13,18^.

Why this Alzheimer’s Society call is the right place to fund our study

The Alzheimer’s Society (AS) has identified ‘Getting a Diagnosis’ as one of the priority “Challenges facing primary carers for people with dementia”^30^. Our study sits alongside the AS campaign to increase diagnosis and adds value at a relatively low cost. The project targets many of the barriers (e.g. patients refusing a referral, techniques to persuade a patient to accept help, relative’s giving history of memory loss, leaflets aimed at family carers) identified in the AS consultation. The Alzheimer’s Society consumer groups, including GPs and carers, are working collaboratively with us, and the AS designers, in developing the intervention from the research base to practice. It engages the AS unique resources of consumers and dementia experts. This joint project fits with the AS research priorities and with its aim to give evidence based advice^31^. By assisting people to recognise dementia and seek appropriate help we are equipping people to play a more active role in their health care. Improvements in health will be demonstrated by the increased uptake of diagnostic services for people with memory problems. This is an innovative and simple change in practice and currently no such approaches are known to have been tried within dementia to encourage an improvement in the number of people with dementia receiving a diagnosis. Professor Burns (Dementia “Tsar”) indicates that the Department of Health will support adopting the intervention for wider dissemination if it is successful (“120%”).

**3. Detailed experimental design and methods to be used in investigating the problem**

The study will be a pragmatic, multi-site, cluster randomised controlled trial in which the intervention and treatment as usual group will be compared in terms of cognitive severity at diagnosis. The intervention is a personalised letter from their GP and leaflet (see figure 1 below). The leaflet contains information about overcoming common barriers to accessing dementia diagnosis care if people are concerned about a relative or themselves^1^.

It covers:

- How to persuade someone to go to the doctor if they or their family are worried about their memory.
- What to do if the GP can’t see there is a problem
- What to do to be referred to specialist services
- What to do to prevent the doctor being unable to talk to you about your relative because of confidentiality
- What to do if the person with memory problems refuses help
- What information patients and families can get about the illness
- What to do if things are not working
- Help available for carers

**4. Study population**

We will include all registered patients within GP practices over the age of 70 years who do not currently have a diagnosis of dementia, live in a care home or have been referred to specialist memory services.


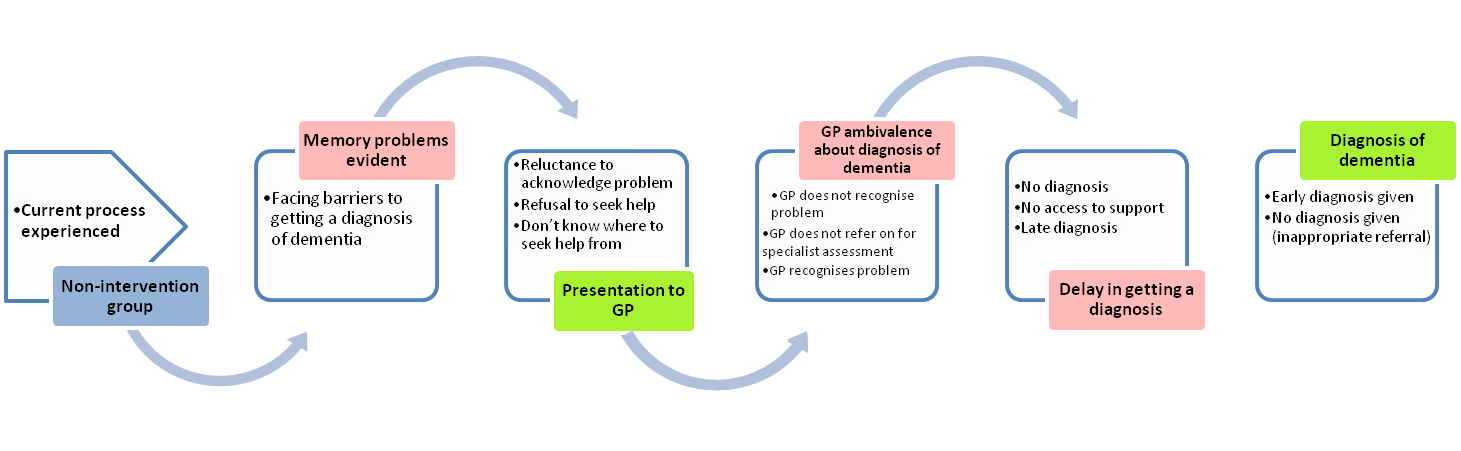


KEY

Problems experienced

Outcomes measured


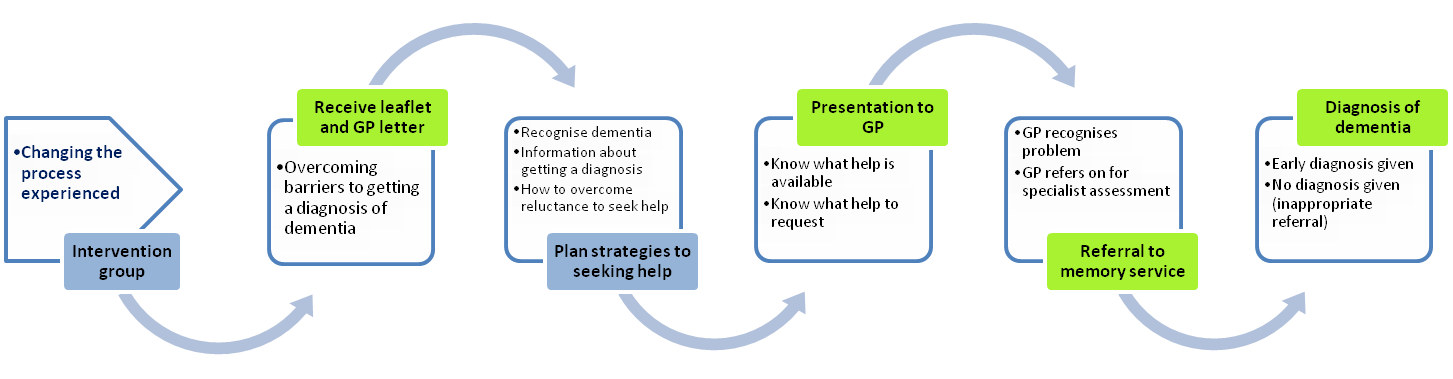


**Referral to memory service**

**Figure 1 Project plan for facilitating early diagnosis in dementia**

**5. Recruitment sites**

The research will take place in UCLPartners partnership trusts where our team work clinically. The partnership consists of UCL and the local primary and secondary care trusts including mental health trusts: Camden and Islington NHS Foundation Trust, North East London NHS Foundation Trust, North Essex Partnership NHS Foundation Trust and Barnet Enfield and Haringey Mental Health Trusts. Prof Orrell is the dementia lead for UCLPartners and a collaborator on the project. UCLPartners have agreed collaboration and the sites are geographically accessible. The project fits with UCLPartners’ dementia strategy with which all participating trusts are engaged. UCLPartners will help facilitate the engagement of commissioners to fund additional activity within the memory services if necessary. We will also approach surrounding counties to London to recruit GP practices if needed, such as Hertfordshire, Essex, Kent, Surrey and Berkshire. Sites are diverse in socioeconomic status, ethnic composition, and are inner-city to rural. This covers the variation within England enabling our results to be externally valid. Prof de Lusignan a co-applicant is a research active GP as is Dr Rait who is a collaborator. The recruitment of GP practices will be facilitated through Dementia and Neurodegenerative Diseases (DeNDRoN), the Primary Care Research Networks (PCRN) and Alzheimer’s Society GP Reference Group.

**6. Patient, service and public involvement**

A family carer from the Alzheimer's Society Research Network helped develop the protocol and she will be a member of the Trial Steering committee. We will consult further on the design and content with the Alzheimer’s Society GP reference group and through focus groups with Alzheimer’s Society including dementia advisors; dementia patients and carers, and consult iteratively about changes in the materials from September 2012 over the following five months. An Admiral Nurse, representing the voluntary sector organisation Dementia UK is a collaborator and will be a member of the project management committee. Family carers were the source of all information on the CHOICE leaflet and helped write it.

**7. Procedure (see Figure 2 Gantt chart timeline)**

We will begin by recruiting GP practices from the intervention and finalise Research and Development approval (explained fully in ethics section). We already have three local practices and two memory services engaged. Dr Hoe will visit clinical settings individually to discuss the study. We will start to engage commissioners and recruit more memory services, asking them to plan to make this intervention if successful part of usual practice. We will discuss the possibility of extra workload requiring funding although this should be small for any individual clinic.

Recruitment will continue as the pragmatic, multi-site, cluster randomised controlled trial begins. Clustering will be by practices. We will send the intervention to people aged over 70 years in the included practices. Many or most of those will either know someone with a diagnosis of dementia or themselves have symptoms. We will exclude those with an existing diagnosis of dementia or referral to memory services, and those living in care homes (as most will already have a diagnosis). The intervention’s effectiveness will be evaluated using routinely collected data. Data will be collated at baseline, 3, 6 and 12 months after the letters are sent. The leaflets are available on the internet and we will not try to influence GPs use of them in the usual practice group.

**8. Data collection**

All data will be anonymised and collected for intervention and control practices. Consent will be through cluster guardians^32^, who will provide consent for collection of this data^33^.

1. Sociodemographic data about the person with suspected dementia will include gender, age, ethnic status according to census, marital status, education, living situation and location. We will record GP characteristics and practice size.
2. **The primary outcome** is the cognitive score as measured by the Mini Mental State Examination (MMSE)^34^ of people referred to memory services in the intervention and control groups who receive a diagnosis of dementia. The minimum clinically important difference in MMSE score is three points^2^ and is the most widely used standard test of cognitive function^34^. We will collate the baseline MMSE score from the new patients’ presenting to memory clinic (i.e. MMSE scores of new presentations) at 3, 6 and 12 months after sending the letters. We are not collecting follow-up MMSEs. There is a maximum score of 30, with scores of 0-10=severe, 11-20=moderate, and 21-24=mild dementia^35^.
3. **Secondary outcomes**

- Number of people presenting with a possible diagnosis of dementia will be collected from GPs practices using MiQUEST^36^ (a Department of Health computer system allowing anonymised data extraction from GP practices). Prof de Lusignan is an expert in using this and is one of the project team. We will collect the number of people presenting to their GP who are and who are not referred on to memory services.
- Numbers of patients referred to memory services from participating intervention and control practices
- The number of memory services appointments offered and people attending for diagnostic assessment,
- The number of people referred who do not have dementia or mild cognitive impairment
- Costs of implementing the intervention, including the costs associated with the CHOICE leaflet (printing, and distribution), GP visits to discuss possible diagnoses of dementia, and use of memory services, and the cost of assessing those with and without dementia
- We will systematically ask clinicians about any inappropriate presentations and patient distress and record these as adverse events.

**9. Power calculations and statistical methods to be used**

Sample size

Approximately 14% (1 in 7) of people aged >70 have dementia^37^. The average GP registered patient population is ≈2000^38^, of whom 260 (13%)^39^ are ≥70 years old. Thus there is a mean of 36-37 patients with dementia per GP of whom 24 would be unknown. An audit of people with dementia presenting to Islington memory services showed a mean MMSE score =19.5 (standard deviation=6.1). A clinically significant difference between experimental and control arms =3 points in MMSE^2^. To achieve this would require 71 undiagnosed people with dementia in each group (α=5% and power=90%). In Islington last year 420 patients were referred from the 38 practices – a mean of 11/practice. Since families in group practices may receive the intervention letter from one GP then consult and be referred by another, cluster randomisation is appropriate and will be used to allocate GP practices to the intervention or control arms. Consequently, we need to inflate the sample based on a projected intracluster correlation of 0.03^40^. The required sample size in each arm is then 93. This will equate to 8580 people aged over 70 registered with the GPs. If 11 patients are referred per practice, we require approximately nine practices (clusters) in each trial arm. The primary outcome is routinely collected data; but some patients will become too ill or die, or refuse assessment by the clinics. Estimated level of attrition rate=18% (18% loss to follow up in LASER including deaths- a previous dementia study in the community), and therefore we aim to recruit 11 GP practices in each arm. Randomisation will be computerised and stratified by geographical location. The allocation ratio will be 1:1.

**10. Analysis**

We will report descriptive sociodemographic and clinical data by randomisation group. We will conduct between group comparisons for categorical and linear data. We will compare the mean MMSE from each group and will use t-tests or Mann-Whitney where appropriate. Statistical modelling will be performed in the form of hierarchical multivariate regression analysis, to account for clustering of patients. Analysis of covariance (ANCOVA) will be used to adjust for baseline differences in outcome variables and multiple linear regression will be used to identify other predictors of MMSE at presentation e.g. gender, age and ethnicity. In the regression analysis we will account for the clustering effect in suitable ways (e.g. random effects models). We plan to perform a primary analysis on complete cases and then develop secondary analyses adjusting for potential missing data using suitable methods (e.g. multiple imputations, and inverse probability weighting). We will apply these as sensitivity analysis and discuss them in conjunction with the main analysis. Missing data is accounted for in the sample size calculations; thus we should be able to observe enough complete cases to preserve the power for the main analysis. For the cost analysis the costs associated with printing and distributing the CHOICE leaflet will be based on market prices. Numbers of GP visits will be taken from MiQUEST^29^ and numbers of contacts with memory services will be taken from practice records. All contacts will then be costed by applying unit costs from published sources^41^. We will then calculate and compare the mean cost of delivering the intervention per patient and the use of GP and memory services in each arm.

**Figure 2: Milestones and project timetable**

| **Gantt chart** | | | | | | | | | | | | |
| --- | --- | --- | --- | --- | --- | --- | --- | --- | --- | --- | --- | --- |
| **Project Timescale -** (30 months)  To commence – 01/02/13  To complete – 31/07/13 | | | | | | | | | | | | |
| Year | 2012 | 2013 | | | | 2014 | | | | 2015 | | |
| Quarter |  | 1 | 2 | 3 | 4 | 1 | 2 | 3 | 4 | 1 | 2 | 3 |
| Application | **x** |  |  |  |  |  |  |  |  |  |  |  |
| Submission | **x** |  |  |  |  |  |  |  |  |  |  |  |
| Awarded | **x** |  |  |  |  |  |  |  |  |  |  |  |
| Commences |  | **x** |  |  |  |  |  |  |  |  |  |  |
| **Project planning & preparation** | | | | | | | | | | | | |
| Develop Trial Master File |  | **x** |  |  |  |  |  |  |  |  |  |  |
| Write & submit ethics application | **x** | **x** |  |  |  |  |  |  |  |  |  |  |
| Obtain R&D approvals |  | **x** | x |  |  |  |  |  |  |  |  |  |
| Project awareness raising |  | **x** |  |  |  |  |  |  |  |  |  |  |
| **Meetings** | | | | | | | | | | | | |
| Research Meetings | **x** | **x** | **x** | **x** | **x** | **x** | **x** | **x** | **x** | **x** |  |  |
| Trial Management group |  | **x** |  | **x** |  | **x** |  | **x** |  | **x** |  |  |
| Trial Steering committee |  | **x** |  | **x** |  | **x** |  | **x** |  | **x** |  |  |
| **Starting the study** | | | | | | | | | | | | |
| Recruitment – GP practices & Memory Services |  | **x** | **x** | **x** |  |  |  |  |  |  |  |  |
| Engage commissioners |  | **x** | **x** |  |  |  |  |  |  |  |  |  |
| Visit GP practices & Memory Services |  | **x** | **x** | **x** | **x** |  |  |  |  |  |  |  |
| **Delivering the trial** | | | | | | | | | | | | |
| Randomise 22 GP practices to intervention & control groups |  |  | **x** | **x** | **x** |  |  |  |  |  |  |  |
| Collect baseline data |  |  | **x** | **x** | **x** |  |  |  |  |  |  |  |
| Deliver Trial interventions |  |  | **x** | **x** | **x** |  |  |  |  |  |  |  |
| Collect follow up data at 3,6,12 months |  |  | **x** | **x** | **x** | **x** | **x** | **x** | **x** |  |  |  |
| Data entry |  |  | **x** | **x** | **x** | **x** | **x** | **x** | **x** |  |  |  |
| Data Analysis |  |  |  |  |  |  |  | **x** | **x** | **x** |  |  |
| Determine cost benefits |  |  |  |  |  |  |  | **x** | **x** | **x** |  |  |
| **Dissemination** | | | | | | | | | | | | |
| Write up results for publication |  |  |  |  |  |  |  |  | **x** | **x** | **x** | **x** |
| Present findings |  |  |  |  |  |  |  |  |  | **x** | **x** | **x** |
| Commissioners |  |  |  |  |  |  |  |  |  | x | x | x |

**11. Dissemination plans**

We will actively disseminate best practice and knowledge, to implement the intervention. One key enabler of diffusion is the innovation process undertaken with testing, leading to ownership by AS and partners. We will engage our network of partners (e.g. Alzheimer’s Society, Dementia UK, UCL, UCLPartners, DeNDRoN, commissioners and DH) from the outset as a ‘community of interest’ throughout to ensure acceptability and as a diffusion network beyond the test sites. In particular, we will explore how we might link with voluntary organisations’ networks to explore the potential of diffusing this innovation through ‘patient or carer-pull’. Innovations considered by adopting organisations to be observable, modifiable and non-complex are easier to diffuse^42^. We will present the findings locally, nationally and internationally. The standard letter and final leaflet will be published and made widely available. The findings will also be disseminated through research papers in peer review journals. Our dissemination will include a focus on a project which is a voluntary sector/university/state partnership and on the simple, tangible and adaptable nature of the intervention.

**12. Data transfer (handling, processing and storage)**

In the study, data as described in section 8 of this protocol will be collected from patient records in accordance with the GP and memory service cluster guardian consent forms, patient information sheet and section 8 of this protocol. The sociodemographic and clinical outcomes data will be appropriately sent to Dr Juanita Hoe, University College London for data entry and statistical analysis and Dave Wilson, Joint Research Office, UCL will act as the data controller of such data for the study.

Dr Juanita Hoe, will process, store and dispose of the data collected as part of the FED_D study in accordance with all applicable legal and regulatory requirements, including the Data Protection Act 1998 and any amendments thereto. Some of the data will be collected using paper and pens and inputted into an electronic database or software package on NHS or university computers. All computers will be password protected. Data collected on paper records will be stored in a locked cabinet in a locked room at UCL and accessed only by the research team.

**13. UCL Insurance and Indemnity Section**

University College London holds insurance against claims from participants for harm caused by their participation in this clinical study*.* Participants may be able to claim compensation if they can prove that University College London has been negligent. However, if this clinical study is being carried out in a hospital, the hospital continues to have a duty of care to the participant of the clinical study. University College London does not accept liability for any breach in the hospital’s duty of care, or any negligence on the part of hospital employees. This applies whether the hospital is an NHS Trust or otherwise.

**14. Archiving**

University College London and each participating site recognise that there is an obligation to archive study-related documents at the end of the study (end of December 2015). The Chief Investigator confirms that he/she will archive the study master file at University College London for 20 years from the study end. The Principal Investigator at each participating site agrees to archive his/her respective site’s study documents for 5 years from the study end.

**Appendix:** CHOICE Information Leaflet

**References**

1. **Livingston G, Leavey G, Manela M, Livingston D, Rait G, Sampson E, Bavishi S, Shahriyarmolki K, Cooper C** (2010) Making decisions for people with dementia who lack capacity: qualitative study of family carers in UK. *BMJ.*18;341:c4184. doi:10.1136/bmj.c4184.
2. **Burback D, Molnar FJ, St John P, Man-Son-Hing M** (1999). Key methodological features of RCTS of Alzheimer’s disease therapy: minimal clinical difference, sample size and trial duration. Dement Geriatr Cogn Disord 10:534–540.
3. **Department of Health, Care Services Improvement Partnership** (2005) *Everybody’s Business. Integrated Mental Health Services for Older Adults: A Service Development Guide*. The Stationery Office, London.
4. **Luengo-Fernandez R, Leal J, Gray A** (2010) *Dementia 2010: The prevalence, economic cost and research funding of dementia compared with other major diseases.* Alzheimer’s Research Trust, Cambridge.
5. **Ferri, C.P. Prince,M. Brayne,C. Brodaty, H. Fratiglioni, L. Ganguli, M. Hall, K. Hasegawa, K. Hendrie, H. Huang, Y. Jorm, A. Mathers, C. Menezes, P.R. Rimmer, E. Scazufca, M. For Alzheimer’s Disease International** (2005) Global prevalence of dementia: a Delphi consensus study’ *Lancet*, 366:9503:2112-2117.
6. **Knapp M, Prince M, Albanese E, Banerjee S, Dhanasiri S, Fernandez J, Ferri C, Knapp M, McCrone P, Prince M, Snell T, Stewart R** (2007) *Dementia UK. The Full Report*. Alzheimer’s Society, London.

URL: <http://www.nao.org.uk/publications/nao_reports/06-07/0607604.pdf>

1. **Department of Health** (2009) *Living Well with Dementia: A National Dementia Strategy*. The Stationery Office, London.
2. **National Audit Office** (2010) *Improving Dementia Services in England – an Interim Report*. *GP Survey results*. The Stationery Office, London.
3. **Olafsdottir M, Foldevi M, Marcusson** **J** (2001). Dementia in primary care: Why the low detection rate? *Scandinavian Journal of Primary Health Care*. 19:3:s.194-198.

doi:10.1080/028134301316982469 URL: <http://dx.doi.org/10.1080>

1. **National Audit Office** (2007) *Improving Services and Support for People with Dementia*. The Stationery Office, London.

URL: <http://www.nao.org.uk/publications/nao_reports/06-07/0607604.pdf>

1. **Teel, C, & Carson P**. (2003). Family experiences in the journey through dementia diagnosis and care. *Journal of Family Nursing*, 9:1:38–58.

doi:10.1177/1074840702239490

1. **Robinson A, Elder J, Emden C, Lea E, Turner P, Vickers J** (2009) Information pathways into dementia care services: Family carers have their say. *Dementia*, 8:17-37. doi:10.1177/1471301208099051
2. **Banerjee S, & Wittenberg R (2009)** Clinical and cost effectiveness of services for early diagnosis and intervention in dementia. *International Journal of Geriatric Psychiatry.* 24:7:748-754. DOI:10.1002/gps.2191
3. Gilley DW, Wilson RS, Bienias JL, Bennett DA, Evans DA (2004) Predictors of
   depressive symptoms in persons with AD. *Journal of Gerontology.* 59B:75–83.
4. Yaffe K, Fox P, Newcomer R, Sands L, Lindquist K, Dane K, Covinsky KE (2002) Patient and caregiver characteristics and nursing home placement in patients with dementia. *Journal American Medical Association*, 287:16:2090-2097
5. **All-Party Parliamentary Group on Dementia** (January 2012) URL:<https://www.alzheimers.org.uk/site/scripts/documents_info.php?documentID=1583&pageNumber=2>
6. **Wright J** (2011) <http://dementianews.wordpress.com/2011/08/02/a-misspent-opportunity-all-party-parliamentary-group-on-dementia-2010-alzheimers-society/>
7. **National Audit Office** (2010) *Improving Dementia Services in England – an Interim Report*. The Stationery Office, London.

URL: <http://www.nao.org.uk/publications/0910/improving_dementia_services.aspx>

1. **Alzheimer’s Society** (2011) *Mapping the Dementia Gap*

URL: <http://alzheimers.org.uk/site/scripts/download_info.php?fileID=1058>

1. **Iliffe S, Wilcock J, Haworth D. (2006)** Obstacles to shared care for patients with dementia: a qualitative study. *Family Practice.* 23:3:353-362.

doi:10.1093/fampra/cmi116

1. **Rait G, Walters K, Bottomley C, Petersen I, Iliffe S, Nazareth I.** (2010) Survival of people with clinical diagnosis of dementia in primary care: cohort study. *BMJ*. 341:c3584. doi: 10.1136/bmj.c3584
2. **National Audit Office** (2007) *Improving Services and Support for People with Dementia*. The Stationery Office, London.

URL: <http://www.nao.org.uk/publications/nao_reports/06-07/0607604.pdf>

1. **Kostopoulou O, Delaney BC, Craig W, Munro CW** (2008) Diagnostic difficulty and error in primary care — a systematic review. *Family Practice*. 25:6:400-413. doi:10.1093/fampra/cmn071
2. **Lopponen M, Raiha I, Isoaho R. Vahlberg T, Kivela S-I,** (2003) Diagnosing cognitive impairment and dementia in primary health care – a more active approach is needed. *Age & Ageing.* 32:6:606-612. doi:10.1093/ageing/afg097
3. **Chan T, van Vlymen J, Dhoul N, de Lusignan S** (2011) Using routinely collected data to evaluate a leaflet campaign to increase the presentation of people with memory problems to general practice: a locality based controlled study. *Informatics in Primary Care*, 18:3:189-196(8)
4. **Medical Research Council** (2000) A framework for development and evaluation of RCTs for complex interventions to improve health. London: MRC.
5. **Medical Research Council** (2008) Developing and evaluating complex interventions: new guidance. London: MRC
6. **CHOICE:** DECISION MAKING FACT SHEETS for family/friend carers

URL: <http://www.bmj.com/content/suppl/2010/08/18/bmj.c4184.DC1/livg719658.ww1_default.pdf>

1. **Robb K, Power E, Kralj-Hans I, Edwards R, Vance M, Atkin W, Wardle J** (2010) Flexible sigmoidoscopy screening for colorectal cancer: uptake in a population-based pilot programme. *Journal of Medical Screening.* 17:2:75-78. doi:10.1258/jms.2010.010055
2. **Alzheimer’s Society** (2012) Challenges facing primary carers of people with dementia: Opportunities for research. Alzheimer’s Society, London

<http://www.alzheimers.org.uk/site/scripts/download_info.php?downloadID=840>).

1. **Sorenson S** (2009) *Alzheimer’s Society Research Funding Strategy*. Alzheimer’s Society, London. URL: <http://www.alzheimers.org.uk/site/scripts/documents_info.php?documentID=1111>
2. **Medical Research Council** (2002) *Cluster randomised trials: Methodological and ethical considerations*. MRC clinical trials series. London: MRC.
3. **Mental Capacity Act, 2005** (2007) *Code of Practice*. London:TSO

URL: [http://www.dca.gov.uk/menincap/legis.htm#codeofpractice](http://www.dca.gov.uk/menincap/legis.htm#codeofpractice)

1. **Folstein MF, Folstein SE, McHugh PR** (1975) Mini Mental State. A practical method for grading the cognitive state of patients for the clinician. *Journal of Psychiatric Research*, 12:189-198.
2. **Ashford JW** (2000). *Mini-Mental State Exam with Item-Response Calculation.*
   URL: <http://www.medafile.com/mmsei.htm>
3. **NHS Connecting for Health. *MIQUEST***.

[URL:www.connectingforhealth.nhs.uk/systemsandservices/data/miquest](url:www.connectingforhealth.nhs.uk/systemsandservices/data/miquest)

1. **Plassman BL, Langa KM, Fisher GG, Heeringa SG, Weir DR, Ofstedal MB, Burke JR, Hurd MD, Potter GG, Rodgers WL, Steffens DC, Willis RJ, Wallace RB** (2007) Prevalence of Dementia in the United States: The Aging, Demographics, and Memory Study. *Neuroepidemiology*, 29:125–132

DOI: 10.1159/000109998

1. **Gregory S** (2009) *General practice in England: An overview.* The King’s fund. London.
2. **Office National Statistics** (2010) *Mid-year population estimates*. Office for National Statistics; General Register Office for Scotland; Northern Ireland Statistics and Research Agency. URL: <http://www.statistics.gov.uk/cci/nugget.asp?id=949>
3. **Machin D, Campbell M, Fayers P, Pinol A** (1997) *Sample size tables for clinical studies.* Second Edition. Oxford: Blackwell.
4. **Personal Social Services Research Unit** (2011) Unit Costs of Health & Social Care 2011. PSSRU:University of Kent.
5. **Rogers EM** (2003) *Diffusion of innovations*. New York: Free Press.
