# Supplementary material for: Effectiveness of an intervention to facilitate prompt referral to memory clinics in the United Kingdom: Cluster randomised controlled trial
Source: PLoS Med. 2017 Mar 14;14(3):e1002252. doi: 10.1371/journal.pmed.1002252 (PMC5349651; doi:10.1371/journal.pmed.1002252)
Supplement: S2 Text — (DOC) [file pmed.1002252.s002.doc]

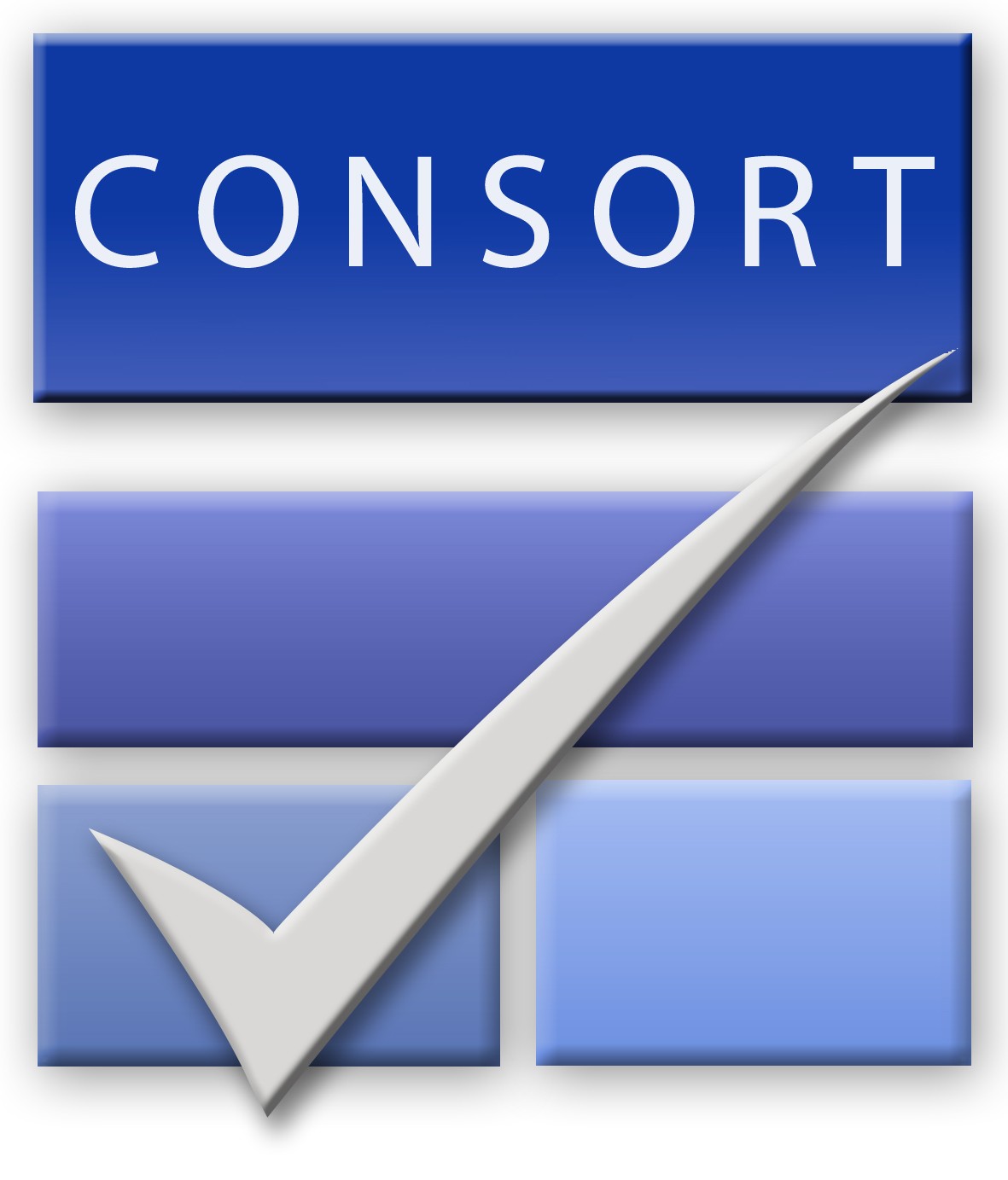
S2 text CONSORT 2010 checklist of information to include when reporting a randomised trial*

| Section/Topic | Item No | Checklist item | Reported on page No |
| --- | --- | --- | --- |
| Title and abstract | | | |
|  | 1a | Identification as a randomised trial in the title | Title |
|  | 1b | Structured summary of trial design, methods, results, and conclusions (for specific guidance see CONSORT for abstracts) | Abstract |
| Introduction4 | | | |
| Background and objectives | 2a | Scientific background and explanation of rationale | Introduction |
|  | 2b | Specific objectives or hypotheses | Introduction –end |
| Methods | | | |
| Trial design | 3a | Description of trial design (such as parallel, factorial) including allocation ratio | Methods -subheading study design |
|  | 3b | Important changes to methods after trial commencement (such as eligibility criteria), with reasons | No changes |
| Participants | 4a | Eligibility criteria for participants | Methods , subheading patients |
|  | 4b | Settings and locations where the data were collected | Methods –subheading study design |
| Interventions | 5 | The interventions for each group with sufficient details to allow replication, including how and when they were actually administered | Methods- subheading procedures, then subheading intervention and treatment as usual |
| Outcomes | 6a | Completely defined pre-specified primary and secondary outcome measures, including how and when they were assessed | Methods – subheading outcomes |
|  | 6b | Any changes to trial outcomes after the trial commenced, with reasons | Methods –subheading analysis, then subheading changes to trial outcome after the trial commenced |
| Sample size | 7a | How sample size was determined | Method- subheading statistical analysis then subheading power calculation |
|  | 7b | When applicable, explanation of any interim analyses and stopping guidelines | none |
| Randomisation: |  |  |  |
| Sequence generation | 8a | Method used to generate the random allocation sequence | Methods- subheading. –randomisationand masking |
|  | 8b | Type of randomisation; details of any restriction (such as blocking and block size) | Methods- subheading. –randomisationand masking |
| Allocation concealment mechanism | 9 | Mechanism used to implement the random allocation sequence (such as sequentially numbered containers), describing any steps taken to conceal the sequence until interventions were assigned | Methods- subheading. –randomisationand masking |
| Implementation | 10 | Who generated the random allocation sequence, who enrolled participants, and who assigned participants to interventions | Methods- subheading. –randomisationand masking |
| Blinding | 11a | If done, who was blinded after assignment to interventions (for example, participants, care providers, those assessing outcomes) and how | Methods- subheading. –randomisationand masking |
|  | 11b | If relevant, description of the similarity of interventions | None |
| Statistical methods | 12a | Statistical methods used to compare groups for primary and secondary outcomes | Methods –statistical analysis-subheading analysis and post-hoc analysis |
|  | 12b | Methods for additional analyses, such as subgroup analyses and adjusted analyses | Methods –statistical analysis-subheading analysis and post-hoc analysis |
| Results | | | |
| Participant flow (a diagram is strongly recommended) | 13a | For each group, the numbers of participants who were randomly assigned, received intended treatment, and were analysed for the primary outcome | Beginning of results section and consort diagram-figure 1 |
|  | 13b | For each group, losses and exclusions after randomisation, together with reasons | Beginning of results section and consort diagram-figure 1  consort diagram |
| Recruitment | 14a | Dates defining the periods of recruitment and follow-up | Beginning of results section |
|  | 14b | Why the trial ended or was stopped | Beginning of results section |
| Baseline data | 15 | A table showing baseline demographic and clinical characteristics for each group | Results-table 1 |
| Numbers analysed | 16 | For each group, number of participants (denominator) included in each analysis and whether the analysis was by original assigned groups | Results |
| Outcomes and estimation | 17a | For each primary and secondary outcome, results for each group, and the estimated effect size and its precision (such as 95% confidence interval) | Results |
|  | 17b | For binary outcomes, presentation of both absolute and relative effect sizes is recommended | Not applicable |
| Ancillary analyses | 18 | Results of any other analyses performed, including subgroup analyses and adjusted analyses, distinguishing pre-specified from exploratory | Results |
| Harms | 19 | All important harms or unintended effects in each group (for specific guidance see CONSORT for harms) | Results text and table 3 |
| Discussion | | | |
| Limitations | 20 | Trial limitations, addressing sources of potential bias, imprecision, and, if relevant, multiplicity of analyses | Discussion-paragraph 6-9 |
| Generalisability | 21 | Generalisability (external validity, applicability) of the trial findings | Clinical implications and conclusions |
| Interpretation | 22 | Interpretation consistent with results, balancing benefits and harms, and considering other relevant evidence | Discussion and clinical implications and conclusion |
| Other information | | |  |
| Registration | 23 | Registration number and name of trial registry | Trial registration |
| Protocol | 24 | Where the full trial protocol can be accessed, if available | Methods than study design |
| Funding | 25 | Sources of funding and other support (such as supply of drugs), role of funders |  |

*We strongly recommend reading this statement in conjunction with the CONSORT 2010 Explanation and Elaboration for important clarifications on all the items. If relevant, we also recommend reading CONSORT extensions for cluster randomised trials, non-inferiority and equivalence trials, non-pharmacological treatments, herbal interventions, and pragmatic trials. Additional extensions are forthcoming: for those and for up to date references relevant to this checklist, see [www.consort-statement.org](http://www.consort-statement.org/).
