# Supplementary material for: Effectiveness of an intervention to facilitate prompt referral to memory clinics in the United Kingdom: Cluster randomised controlled trial
Source: PLoS Med. 2017 Mar 14;14(3):e1002252. doi: 10.1371/journal.pmed.1002252 (PMC5349651; doi:10.1371/journal.pmed.1002252)
Supplement: S3 Text — (PDF) [file pmed.1002252.s003.pdf]

Getting Help for Memory Problems

If you or your relative have a problem with your memory, the first point of contact is your GP.

Occasional lapses in memory are common, particularly as we get older.

Many people with memory problems do not have dementia.

"The assumption is that people who are losing their memory have got dementia coming on, which is not necessarily the case…"
Person with memory problems

"…it is not a stigma, it's not something to be ashamed of."
Person with memory problems

Photograph 1

What can I do if my relative doesn't want to see a doctor to find out what's wrong?

People with memory problems or dementia may be unaware of the situation early on. Families may notice it first.

"I often used to have to apologise for my being absent minded, but it got a lot more serious...as time went on...I forgot some really vital things like appointments and that."
Person with memory problems

"The hardest was to convince my wife there was some-thing wrong, she didn't want to know, she wouldn't talk about it."
Husband

"With hindsight, you know when somebody has developed Alzheimer's for instance, you think back over the years, over the two and three years, oh yes, there's little signs there, but we're not recognising them necessarily."
Person with memory problems

Some families used an appointment with the GP about a separate issue to bring up the memory problems. Or you could ask the GP to write to your relative and offer them an appointment.

"I used to be a bit conniving, say I'm coming to see the doctor, that's the only way I could get her there."
Husband

"He hates going to the doctors… they sent a letter saying that they needed to see him."
Wife

It often helps to go to the GP together.

"My children were talking to me and it was like I didn't understand, I kept asking the same questions 'Oh mummy but we told you'...it took them to say 'mummy...we are taking you to the doctors'... that's when I accepted it."
Person with memory problems

What can I do to help the doctor?

It often takes time for a diagnosis of dementia to be made, which can be very difficult. You can write a list of the problems about you or your relative's memory and functioning for your GP.

"It took me a while to get [the doctor] to give her a test, later my daughter found the gas turned on, so when I left for work I had to turn [it] off, then I really pushed the doctor."
Husband

What can I do to get referred to a specialist (memory clinic)?

The GP may need to do memory and blood tests first. Relative's information is often necessary as someone with memory problems may forget important things.

"Ask for a referral to a specialist, ASAP."
Husband

What information can I get about my illness?

After your diagnosis, you can contact their care team if you are unsure of anything. Many local Alzheimer's Societies run courses and national helplines can send information. The Internet is also valuable (see last page for recommended sites).

"Get as much information as possible [it] is there."
Wife

"..it may help to know what is going to happen in the future."
Person with memory problems

What can I do if the doctor can't talk to me because information about my relative's health is confidential?

Medical confidentiality means doctors cannot give out information. You can go with your relative to see the doctor. Once the GP knows that your relative gives permission to tell you things, they will continue to give you information.

"It's not bad [getting information] with the doctors because the doctors know me. I had to get my mother's permission to represent her."
Daughter

What happens if my relative doesn’t want help?

“I think it helps if you have reassurance first [about what it means].”
Person with memory problems

You can talk to family members about how using services can help keep their independence, and you can introduce them one at a time so your relative can get used to them.

“She kept saying “no, I don’t want [carers]…” She [professional] said “Try and help slowly”. I said “Once a week…”. They started a care package and it is every day now.”
Son

Often people with dementia are reluctant to accept services. It can help to involve a doctor or other professional to persuade your family member.

“He’ll do it for the doctor but not for me.”
Wife

“So long as you say there’s a doctor, she will listen.”
Daughter

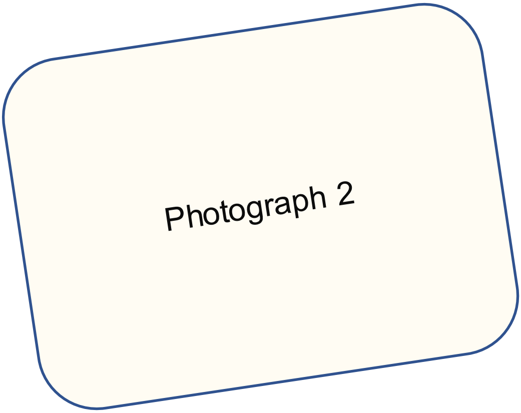

If a service given to your relative is not suitable it can be changed.

What help is available for carers?

Organisations, such as Alzheimer's Society and Age UK, offer services including meeting with others in similar situations.

"Talk to other people about it, see what strategies they have got, but don't try and do it all on your own, you end up then resenting, if you get help you can still love them."
Husband

"I was a member of the local Alzheimer's carers' group and I've really found them enormously helpful."
Friend

You can find out about these organisations online, or ask a professional.

"I asked the nurse, she downloaded lots of information."
Wife

You can ask for advice and help from family and friends as well as voluntary organisations and professional services.

"My advice to other carers would be stop trying to think about it in a logical way because their illness isn't logical."
Husband

"Never be ashamed or embarrassed at asking for help and look within your local community."
Husband

Photograph 3

Things to THINK about - for relatives

- If your relative is reluctant to go to the GP it may help to go together or use an appointment for something else to discuss their memory, or ask the GP to invite them to come in.
- If you go to the GP together, ask your relative in front of the doctor for their permission to be given confidential information about their health.
- If the information after diagnosis seemed overwhelming, you can get information later from the internet or over the phone (see recommended sites overleaf), or discuss things further with your relative's care team.
- Some people with memory problems are reluctant to receive care or admit that anything is wrong. It may help to introduce services slowly, or ask a doctor to explain the need for them.
- You can emphasise that, rather than hindering their independence, care services can increase your relative's quality of life and allow them to live at home comfortably.
- If you are unhappy with your relative's care or treatment, ask for a review.
- You can be offered a carer's assessment to get help for yourself.
- Many services are available for carers, including short breaks if you need a rest.

Photograph 4

Things to THINK about - for people with memory problems

- Make a list of the memory issues for the GP.
- Many people with memory problems do not have dementia.
- Ask if you want the GP to refer you to a memory clinic. Don't wait for a crisis.
- Specialist doctors and nurses will provide copies of their letters to your GP.
- The Internet or national helplines can be a valuable source of information. We have recommended some below. If you prefer to receive printed information, contact the Alzheimer's Society, Admiral Nursing DIRECT or Age UK.
- If you or your relative are concerned about memory problems, information is available on the Alzheimer's Society website, or you can speak to someone on one of the national helplines listed below:

Alzheimer's Society website: http://alzheimers.org.uk/

Alzheimer's Society helpline: Call 0300 222 1122

Admiral Nursing DIRECT: Call 0800 888 6678
or email direct@dementiauk.org

Age UK Advice: Call 0800 678 1174

Thanks to people with memory problems and families who shared their experiences.
