# Supplementary material for: Effectiveness of an intervention to facilitate prompt referral to memory clinics in the United Kingdom: Cluster randomised controlled trial
Source: PLoS Med. 2017 Mar 14;14(3):e1002252. doi: 10.1371/journal.pmed.1002252 (PMC5349651; doi:10.1371/journal.pmed.1002252)
Supplement: S4 Text — (PDF) [file pmed.1002252.s004.pdf]

< Practice address>

< Patient’s address>

< Date>

Dear <Patient’s name>

We are writing to you and other people in our practice, in case you or someone you live with is experiencing memory problems and you want to make an appointment to discuss these.

Occasional lapses in memory are common, particularly as we get older. However, if you or your relative have memory problems that have been **continuing over three to six months**, and are affecting your life you might benefit from medical advice.

Being forgetful can be caused by several things; such as medication, infection, depression and anxiety, or may be an early sign of a condition such as dementia. Common difficulties may include the following:

- **Memory loss** e.g. difficulty recalling information that you have recently heard or read.
- **Finding the right words, or following a conversation** e.g. losing the thread of what you are saying, or being unable to follow a TV programme.
- **Recognising familiar places, people and objects,** e.g. getting lost on familiar routes, or not recognising a relative or old acquaintance.
- **Planning and organising activities** e.g. making appointments or sorting your money.
- **Changes in mood and personality** e.g. feeling anxious or less interested.

We have enclosed a leaflet about situations and concerns that people with memory problems or their families sometimes have. It contains useful advice about overcoming difficulties in gaining help and information for memory problems.

Seeking help for memory problems is important as it may offer reassurance about the concerns you have or allow access to specialist services, support and treatment to help you or your relative.

If you are worried, please make an appointment to see a doctor. It may be helpful to bring this letter with you.

Yours sincerely

< GP name>

General Practitioner
